# Telehealth and Screening Strategies in the Diagnosis and Management of Glaucoma

**DOI:** 10.3390/jcm10163452

**Published:** 2021-08-04

**Authors:** Sze H. Wong, James C. Tsai

**Affiliations:** Department of Ophthalmology, Icahn School of Medicine at Mount Sinai, New York Eye and Ear Infirmary of Mount Sinai, New York, NY 10003, USA; jtsai@nyee.edu

**Keywords:** telehealth, glaucoma, screening, monitoring

## Abstract

Telehealth has become a viable option for glaucoma screening and glaucoma monitoring due to advances in technology. The ability to measure intraocular pressure without an anesthetic and to take optic nerve photographs without pharmacologic pupillary dilation using portable equipment have allowed glaucoma screening programs to generate enough data for assessment. At home, patients can perform visual acuity testing, web-based visual field testing, rebound tonometry, and video visits with the physician to monitor for glaucomatous progression. Artificial intelligence will enhance the accuracy of data interpretation and inspire confidence in popularizing telehealth for glaucoma.

## 1. Introduction

Glaucoma is a progressive disease of the optic nerve and a leading cause of irreversible vision loss. Globally, in 2013, the prevalence of glaucoma was 3.54% among people aged 40–80, affecting 64.3 million [1]. It was estimated that by 2040, this number will increase to 111.8 million [1]. The demand for ophthalmologists to take care of glaucoma patients is expected to exceed the supply. In 2018, the Association of American Medical Colleges (AAMC) forecasted that there will be worsening shortages of physicians in the United States, with an estimated shortfall of 33,800 to 72,700 specialists by 2030 [2]. The report did not state what the estimated shortfall of ophthalmologists will be per se, but the trend is expected to be similar. The reasons for this shortfall include the stagnant number of ophthalmology residency and glaucoma fellowship positions, the increasing number of retiring ophthalmologists, and the aging population. In order to ensure adequate care for the increasing population of glaucoma patients, each ophthalmologist will have to accommodate a greater number of patients, eventually leading to overbooked clinic schedules, long wait times for patients, and crowded waiting rooms. The increasingly long wait times for the next available appointment can be detrimental to patient care. New strategies, such as the use of telehealth, will be increasingly important to limit clinic visits to patients who absolutely need to be seen, without compromising the care of patients with a stable disease.

Telehealth, as defined by Merriam-Webster, is health care provided remotely to a patient in a separate location using a two-way voice and visual communication. A computer or smartphone is needed to establish this communication. Because of the coronavirus disease 2019 (COVID-19) pandemic, the use of telehealth has accelerated due to patients’ fear of contracting COVID-19 and the reduced number of in-person appointments given. Telehealth has also provided a convenient way for people living in rural regions to access their doctors.

There are three main purposes of telehealth in the field of glaucoma. One, is to screen for patients who have glaucoma, or are glaucoma suspects (i.e., those who have optic nerve appearances suspicious but not definitive for glaucoma). Two, for those newly diagnosed with glaucoma, to determine the severity of the disease and treatment plan. Three, for those diagnosed in the past, to monitor for disease progression and change management as needed. Each purpose requires a different set of equipment, as discussed below.

## 2. Equipment

### 2.1. Visual Acuity Test

Visual acuity is checked conventionally with a Snellen chart of letters placed 20 feet or 6 m away. If the patient has a refractive error, one should wear glasses corrected for distance. For each eye, the visual acuity of the smallest line the patient can read (at least half the letters correctly) is recorded. For the patient to perform this at home, one can either purchase a Snellen chart and hang it 20 feet away, print out a Snellen chart online and follow its instructions, or use a smartphone app. Of note, small Snellen charts, such as those on the smartphone, are referenced at reading distance, and would require presbyopic patients (typically those age 40 or above) to wear their reading glasses. A literature review [3] of mobile vision acuity applications revealed that the Peek Acuity application (Peek Vision Ltd., Berkhamsted, England) performed best, with a test–retest variability of ±0.029 Logarithm of the Minimum Angle of Resolution (LogMAR) for 95% confidence interval limits and a mean difference of 0.055 LogMAR when compared with visual acuity measured in clinic.

### 2.2. Intraocular Pressure Measurement

Knowing the intraocular pressure (IOP) is crucial for the diagnosis and management of glaucoma. The Early Manifest Glaucoma Trial (EMGT) demonstrated that reducing the IOP by 25% lowered the risk of glaucoma progression by 50% over 6 years [4]. Measuring IOP via telehealth is a challenge because measurement requires the instillation of an anesthetic eye drop with fluorescein and the use of a Goldmann applanator attached to a slit lamp, which is equipment that can only be used in the clinic by a skilled technician or physician. Portable IOP measuring devices with reasonable accuracy are available for use. In the setting of a glaucoma screening outside of clinic, the Tono-Pen^®^, the Pulsair Air Puff tonometer, the iCare rebound tonometer, the Ocular Response Analyzer, and the Diaton transpalpebral tonometer are suitable devices that technicians can use. At home, patients can rely on the iCare HOME. Intraocular sensors such as the Eyemate^®^ and Injectsense can provide IOP data throughout the day as well. If no equipment is available, the IOP range can be estimated by palpation.

#### 2.2.1. Tono-Pen^®^ (Reichert; Depew, New York, NY, USA)

This is a hand-held electronic device that measures the force needed to applanate the cornea via a plunger. Prior to measurement, a topical anesthetic is applied to the eye, and a sanitized disposable cover is placed over the device tip. The operator then lightly taps the central cornea with the device tip multiple times until 10 measurements are recorded. The average IOP of the 10 measurements, along with a statistical confidence indicator, are displayed. The Tono-Pen^®^ is easy to use and has reasonable accuracy when compared with Goldmann applanation, the gold standard for IOP measurement. A masked, randomized study on 270 eyes showed that Tono-Pen^®^ measurements were 1.7 mm Hg higher than Goldmann applanation for IOPs from 6 to 24 mm Hg [5]. Another study looked at 197 eyes with glaucoma or ocular hypertension and found that Tono-Pen^®^ measurements had a high correlation (*r* ≥ 0.86) with Goldmann applanation. However, at high IOPs (≥30 mm Hg), Tono-Pen^®^ tended to underestimate Goldmann; and at low IOPs (≤9 mm Hg), Tono-Pen^®^ tended to overestimate [6]. Another study [7] of 142 eyes reported a correlation of coefficient of 0.84 between Tono-Pen^®^ and Goldmann measurements. This study likewise subdivided the eyes into IOP ranges. For eyes with low IOPs (4–10 mm Hg), the Tono-Pen^®^ measured an average 1.78 mm Hg higher than Goldmann applanation. For eyes with IOP in the normal range (11–20 mm Hg), the Tono-Pen^®^ measured an average 0.07 mm Hg lower than Goldmann applanation. For eyes with elevated IOPs (21–30 mm Hg), the Tono-Pen^®^ measured an average 1.27 mm Hg lower than Goldman applanation. Additionally, for eyes with very elevated IOPs (31–45 mm Hg), the Tono-Pen^®^ measured an average 4.15 mm Hg lower than Goldmann applanation. The higher the IOP, the more the Tono-Pen^®^ underestimates. Tono-Pen IOP measurements have also been shown to be increased by a greater central corneal thickness (CCT) and a greater corneal resistance factor (CRF) [8,9,10,11,12,13]. However, for most eyes, the Tono-Pen^®^ has reasonable accuracy. The Tono-Pen^®^ is particularly useful for patients with a corneal edema or scar, as Goldmann applanation can underestimate the IOP in the presence of a spongy, edematous cornea and overestimate the IOP in the presence of a calcified scar; the Tono-Pen^®^ is less affected by corneal edema and the device tip can be easily directed away from the scar when measuring. Another distinct of advantage of the Tono-Pen^®^ is that the patient does not need to be upright. If the patient can only remain supine due to a medical condition or cannot position one’s head vertically due to neck or spinal disease, the Tono-Pen^®^ can still be used, as long as the operator ensures that the device tip taps the cornea perpendicularly. Other devices require the head to be upright for accurate measurement.

#### 2.2.2. Air Puff Non-Contact Tonometer

This is a non-contact way to measure IOP. An electric device delivers a puff of air, and the force required to applanate the cornea is recorded. Multiple measurements are taken and the average IOP measured is calculated. Because there is no physical contact to the eye, a topical anesthetic is not required and there is no risk of corneal abrasion or infection from the equipment. The device is automated and easy to use. The noncontact tonometers available on the market include the Pulsair Desktop Tonometer (Keeler; Malvern, PA, USA), the CT-80 (Topcon; Tokyo, Japan), the NT-530/510 (NIDEK; Gamagori, Japan), and the TX-20 (Canon; Tokyo, Japan). Many studies found that noncontact tonometer IOP measurements are in moderate agreement with that of Goldmann applanation; as a result, the authors concluded that air puff tonometers can serve as good screening tools but are not accurate enough to substitute for Goldmann applanation [14,15,16,17,18]. Of note, there is an air puff tonometer that is compact and weighs approximately 2.5 kg called the Pulsair IntelliPuff (Keeler; Malvern, PA, USA). It is portable and can be easily brought to a glaucoma screening venue. Hubanova et al. [19] compared IOP measurements between the IntelliPuff tonometer and Goldmann applanation on 137 eyes and found that there was good agreement with an intraclass correlation coefficient of 0.86. The IntelliPuff tonometer overestimated the IOP by 1.5 ± 1.8 mm Hg in normotensive eyes and 2.3 ± 4.8 mm Hg in hypertensive eyes. Air puff is particularly useful in young children who do not tolerate eye drops well or are particularly anxious, as anesthetic eye drops are not required and there is no tip or probe that contacts the eye. However, some patients do not find the air puffs comfortable and would prefer other methods of measurement.

#### 2.2.3. iCare (iCare Finland Oy; Helsinki, Finland)

This is a hand-held device that bounces a light-weight probe off the cornea. The contact is gentle enough that no topical anesthetic is needed. The IOP displayed is a function of the probe’s deceleration at contact and the contact time, as measured by an induction-based coil system. The first generation TA01i model and the second generation ic100 model require the patient to be upright for measurement. Nakakura et al. [20] compared measurements of the iCare TA01i, iCare ic100, and Goldmann applanation on 106 eyes, and found that both iCare models measured significantly lower IOPs than Goldmann applanation (12.2 ± 2.9, 11.7 ± 3.0, and 16.9 ± 3.2 mm Hg, respectively). Furthermore, both iCare models’ IOP measurements were correlated with central corneal thickness (*r* = 0.50). In contrast, Gao et al. [21] compared TA01i iCare measurements to that of Goldmann applanation on 672 eyes and found no significant differences between the two (18.30 ± 5.10 and 18.52 ± 4.46 mm Hg, respectively; *p* = 0.19), with a correlation coefficient *r* = 0.806. However, for eyes with IOP ≥ 23 mm Hg by Goldmann applanation, the iCare measurements were significantly lower (1.66 mm Hg, *p* = 0.007) than that of Goldmann applanation. Central corneal thickness had a stronger correlation with iCare measurements (*r* = 0.39) than with Goldmann applanation (*r* = 0.19). Subramaniam et al. [22] compared IOP measurements of iCare ic100 with Goldmann applanation in 1000 eyes and reported an intraclass correlation coefficient of 0.73. The ic100 measurements were significantly lower than Goldmann applanation measurements (12.1 vs. 16.2 mm Hg), even when the data were subdivided into different ranges of IOP. In January 2020, the iCare ic200 model was granted marketing authorization in the United States; it allows for IOP measurement even when the patient is reclined or supine. Badakere et al. [23] compared the ic200 with Goldmann applanation in 156 eyes and found that the ic200 readings were on average 1.27 mm Hg higher, but with no statistically significant difference.

A unique benefit of a portable tonometer that does not require a topical anesthetic is that home measurements can be performed. The iCare HOME is a device that allows for easy self-measurement. After loading a single-use probe, the device is placed in front of the eye at an appropriate distance (adjustable with the device’s forehead and cheek supports). A hold of a button allows for six consecutive measurements, and the average measurement, along with the time of measurement, are saved in the device. When the patient returns the device to the clinic, these measurement data can be extracted and a diurnal IOP graph can be generated. Being able to take multiple measurements throughout the day at home is particularly useful in glaucoma patients with disease progression than normal IOPs measured in clinic. In this scenario, glaucoma specialists must determine whether the disease progression is a result of the “normal IOP” measured in clinic being above the target IOP to halt progression, or whether there are IOP elevations not detected because they occurred outside of clinic hours. The iCare HOME is a useful device that can answer this question. A comparison [24] of the iCare HOME measurement by the patient versus Goldmann applanation reported a high correlation (*r* = 0.846); the iCare HOME on average measured 0.70 mm Hg greater than Goldmann applanation (*p* < 0.001), and this difference increased by 1.2% for every 10% increase in central corneal thickness. Importantly, 98% of the 128 participants were able to use the iCare HOME.

#### 2.2.4. Ocular Response Analyzer (Reichert; Depew, NY, USA)

This is a desktop device that uses a stream of air to applanate the cornea. No topical anesthetic is needed. Infrared light is emitted onto the cornea, and an infrared light detector measures a peak in light intensity when the cornea is flat. At this state, the inward applanation pressure is measured. The force of air then increases so that the cornea becomes concave, and then decreases until the cornea is flat again. At this state, the outward applanation pressure is measured. The entire measurement process takes about 20 milliseconds. The inward applanation pressure is greater than the outward applanation pressure, and this difference is the biomechanical property of the cornea termed corneal hysteresis. The device displays the inward intraocular pressure measurement (which should be identical to Goldmann tonometry) and the intraocular pressure measurement corrected by corneal hysteresis. Ehrlich et al. [25] and Ogbuehi et al. [26] compared ocular response analyzer (ORA) IOP measurements with those of Goldmann tonometry and found no statistically significant difference between them. However, a number of studies [27,28,29,30,31] found that the ORA significantly overestimated IOP when compared with Goldmann applanation. The importance of the ORA lies in its ability to measure corneal hysteresis, which is a known risk factor for glaucoma progression [32]. Eyes with a corneal hysteresis < 10 are 2.9 times more likely to have moderate to severe glaucoma than eyes with a corneal hysteresis ≥ 10; thus, corneal hysteresis can serve as a screening tool for glaucoma [33].

#### 2.2.5. Sensimed Triggerfish^®^ Contact Lens (Sensimed; Lausanne, Switzerland)

This soft contact lens, approved by the United States Food and Drug Administration (FDA), has a sensor that takes automated recordings for 24 h of the corneoscleral junction’s dimensional changes, which are thought to correlate with changes in IOP. The lens is composed of silicone and has a high oxygen transmissibility to prevent hypoxia of the cornea. The sensor transmits data wirelessly to a circular antenna taped to the periorbital region. The antenna then sends the data via a cable to a recorder that the user wears hanging from the neck. The contact lens has been shown to be safe and well-tolerated, with a fair amount of reproducibility in diurnal IOP patterns [34]. A clinical trial [35] of 33 patients compared the slope of IOP increase from wake to sleep position measured by the contact lens sensor in one eye versus that of which was measured by the pneumatonometer in the contralateral eye; there was a high correlation coefficient of 0.914, suggesting that the contact lens sensor is accurate in detecting IOP changes.

A unique advantage of using a contact lens is it allows for the generation of a diurnal curve, even when the user is sleeping. This device can detect IOP elevations outside of clinic hours that may provide clues as to why a patient’s glaucoma is progressing despite normal IOPs measured in clinic. In fact, a multicenter study that included 445 patients showed that certain variables measured by the contact lens, such as the night bursts ocular pulse frequency standard deviation and night bursts ocular pulse amplitude standard deviation correlated with prior rates of visual field progression [36].

#### 2.2.6. Other Contact Lenses in Development

Researchers in South Korea developed a soft contact lens that measures IOP using a strain sensor [37]. The contact lens was tested on rabbit and human eyes, and it demonstrated reliable and accurate IOP measurements. Different from Triggerfish^®^, this contact lens sends data wirelessly to a smartphone; thus, allowing for the real-time monitoring of IOP and eliminating the need to carry a bulky recording device.

#### 2.2.7. Eyemate^®^ (Implandata Ophthalmic Products GmbH; Hannover, Germany)

This is a CE-certified IOP sensor placed into the ciliary sulcus during cataract surgery or Boston keratoprosthesis Type 1 (BI-KPro) implantation. Similarly to an intraocular lens, the sensor is foldable and can be injected into the eye through a corneal incision. This 11.2 mm wide silicone implant consists of eight pressure-sensitive capacitors in a single application-specific integrated circuit and a microcoil antenna arranged circumferentially. In order to obtain IOP measurements, a handheld reader device is placed at a short distance in front of the eye. The device emits a high frequency field that powers the sensor, and <2 s is needed for the sensor to measure the IOP and send the data to the reader device. A clinical trial demonstrated the successful implantation of Eyemate^®^ in six patients; pupillary distortion and pigment dispersion were observed and some IOP measurements were significantly different from that of Goldmann applanation [38]. Another clinical trial involved 12 patients who underwent BI-KPro surgery and Eyemate^®^ implantation; IOP measurements were found to correlate with surgical manometry (*r* = 0.87) with a mean difference of 3.9 ± 8.6 mm Hg [39]. The Eyemate^®^ intraocular sensor is the first of its kind and can potentially revolutionize IOP monitoring for post cataract surgery or post BI-KPro surgery patients.

#### 2.2.8. Injectsense (Injectsense, Inc.; Emeryville, CA, USA)

This is an IOP sensor, smaller than a grain of rice, that can be implanted transsclerally via an injection. Similarly to an intravitreal injection, implantation of the sensor can be performed in clinic using an injector that pierces the sclera and pars plana. The device self-anchors in the sclera and acts as a plug to prevent the egress of vitreous humor. The sensor measures the IOP at preset time intervals and stores the data. The patient is instructed to wear a pair of smart glasses once a week in order to recharge the sensor and download the stored IOP data, which are then automatically uploaded to a physician-accessible cloud database. This device is limited to investigational use at this time.

#### 2.2.9. Diaton Transpalpebral Tonometer (DevelopAll Inc.; New York, NY, USA)

This is a digital device that measures IOP through the upper eyelid without contact with the cornea. The patient lies in a recumbent position looking up at a 45-degree angle. A measurer pulls up the upper eyelid so that the lid margin is at the corneal limbus. The tonometer tip is placed perpendicular to the eyelid and parallel to the lid margin. A measurement is conducted when the tonometer tip touches the eyelid. The advantages of this device are measurements that can be performed by anyone after a brief training session, a topical anesthetic is not needed and it causes minimal patient discomfort. When compared with Goldmann applanation, Diaton demonstrated a moderate correlation acceptable for glaucoma screening but not as a substitute for Goldmann applanation in the management of glaucoma patients [40,41,42,43].

#### 2.2.10. Finger Palpation

A crude method for the patient to estimate IOP is via finger palpation on the eye through the upper eyelid and describe whether the eye feels such as a tomato (low IOP), grape (normal IOP), or apple (high IOP). This is especially useful for patients who had recent glaucoma surgery and may experience extremes in IOP. An abnormally soft or firm eye would usually require a visit to the clinic soon.

### 2.3. Anterior Segment Photography

Although photography does not offer as much clarity as an in-person examination on the slit lamp, it can provide important information relevant to the diagnosis of the type of glaucoma and to monitor for postoperative complications. For example, the camera may capture the presence of white material along with pupillary margin, indicative of pseudoexfoliation, or an opacity within the pupil, indicative of a dense cataract.

In the setting of a screening program where a slit lamp is not available, a digital single-lens reflex camera (DSLR) with maximum zoom can be used to capture images. For those who had glaucoma surgery, of which the surgical site is in the superior or inferior conjunctiva, the technician would need to shift the eyelid away and have the patient look at the opposite direction to capture images of these areas. The advantages of the DSLR camera include its high resolution, wide range of magnification, and the ability to adjust the flash intensity. Tweaking the settings allow for high quality images of the anterior segment. It has even been demonstrated that when a DSLR camera’s infrared-blocking filter is replaced with a piece of glass, iris transillumination defects of the iris can be photographed clearly [44].

At home, a cell phone camera can be used to capture a gross image of the eye, but the resolution and magnification are not high enough to visualize the anterior chamber. A smartphone adapter attached over the camera is needed for adequate magnification and near focusing in order to obtain clinically useful images. One such adapter is the Paxos Scope (DigiSight Technologies; San Francisco, CA, USA) which was found to be easy to use and was able to image a variety of anterior segment pathologies [45].

### 2.4. Iridocorneal Angle Imaging

To identify whether a patient is at risk for angle closure glaucoma, the ophthalmologist performs an examination technique called gonioscopy, in which a lens with side mirrors is placed on the cornea. The mirrors allow for the visualization of the anterior chamber angle, which contains the trabecular meshwork, the start of the aqueous humor’s drainage pathway. When a large part of the trabecular meshwork is not visible due to the steepening of the iris, the patient is considered high risk for angle closure, and peripheral laser iridotomy is recommended.

In a setting where an ophthalmologist is not present to perform the gonioscopy, an anterior segment optical coherence tomography (ASOCT) can be used to measure the iridocorneal angle. Optical coherence tomography (OCT) uses near-infrared light to capture a high-resolution cross-sectional image of biologic tissue by the principal of interferometry. ASOCT parameters associated with angle closure include smaller anterior chamber dimensions (width, area, and volume) [46,47], a greater iris thickness and area [48], and a larger lens vault [49,50]. A regression model consisting of these parameters can diagnose angle closure with an area under the receiver operating characteristic curve (AUC) > 0.95 [51]. A validated scoring system can be incorporated into the ASOCT image analysis software to identify eyes with angle closure [52]. If a suspiciously narrow iridocorneal angle is seen in a screening program, pharmacologic pupillary dilation should be deferred (as dilation can induce acute angle closure glaucoma) and the patient should be sent to an ophthalmologist for a gonioscopy to verify the diagnosis. Of note, ASOCT is a desktop device that is not portable and is only available in the clinic setting.

Ultrasound biomicroscopy (UBM) uses a high frequency transducer (35–100 MHz) to obtain high resolution images of the anterior segment and measurements of the anterior chamber depth, angle, and the lens vault. Compared with the ASOCT, UBM has a greater penetration range, allowing for the visualization of the ciliary body. This increased range is particularly useful to visualize the plateau iris, cyclodialysis cleft, anterior choroidal effusions, or any masses beneath the iris. The disadvantage of the UBM is a transmission medium, such as a bag of fluid or gel, between the ultrasound probe and the eye is needed. In order to obtain clinically useful images, the technician needs to be well-trained.

A direct visualization of the iridocorneal angle can be performed using a gonioscopy camera. The Gonioscope GS-1 (NIDEK; Gamagori, Japan) is a desktop device that acquires 360-degree color photographs of the angle. The patient’s eye is anesthetized and coated with gel, and the device’s attached 16-mirror gonioprism contacts the cornea. The system then automatically captures images at different focus points from each gonioprism mirror; it takes approximately 1.5 min to photograph both eyes. Images in focus are then selected and a 360-degree fused panoramic image of the angle can be generated. A more portable gonioscope is the EyeCam (Clarity Medical Systems; Pleasanton, CA, USA), which consists of a handheld camera that can be used to visualize the angle when the probe is held against the eye coated with a coupling gel [53]. The Nanyang Technological University and Singapore Eye Research Institute similarly developed the GonioPEN, a smaller pen-like probe that can obtain images of the angle [54].

### 2.5. Fundus Photography

When evaluating a patient for glaucoma, being able to visualize the optic nerve head is key. Traditionally, photographs of the optic nerve head can only be taken with a desktop camera after the pupil is pharmacologically dilated. In recent years, handheld cameras have been developed that can take fundus photographs, even without dilation. In addition to glaucomatous nerve damage, these cameras allow for the diagnosis of macular pathology and other retinal diseases as well. The portable fundus cameras on the market include the Pictor Prestige (Volk; Mentor, OH, USA), VISUSCOUT^®^ 100 (Zeiss; Oberkochen, Germany), SIGNAL (Topcon; Tokyo, Japan), and VERSACAM^TM^ α (NIDEK; Tokyo, Japan). They are generally gun-shaped with a base charger, weighs approximately 1 pound, has a 40 to 50-degree fundus angle of view (with a pupil at least 3 mm in diameter), has autofocus or manual focus modes, has a touchscreen that displays the photograph taken, and has Wi-Fi connectivity to send images online.

An alternative to using the fundus camera is to rely on a smartphone camera attachment. The D-Eye Retinal Camera (D-EYE Srl, Padova, Italy) is an attachment that can obtain images of the optic nerve, even without pupillary dilation. The camera attachment requires the smartphone to be as close to the eye as possible, and the smartphone’s video mode is turned on so that the split-second frame of which the optic nerve is centered within the field of view and is in focus is captured. This method of obtaining optic nerve photographs requires practice and an adequately large undilated pupil. The image resolution is limited by the smartphone camera. A prospective study demonstrated that vertical cup/disc ratios graded from D-Eye smartphone ophthalmoscopy agreed with those from slit lamp examination in 72.4% of primary open angle glaucoma patients and 66.7% of ocular hypertension patients [55]. Another study revealed that pharmacologic pupillary dilation improved D-Eye’s vertical cup/disc ratio measurement significantly [56].

### 2.6. Optical Coherence Tomography of the Retinal Nerve Fiber Layer

In the past, serial optic nerve head photography was the only way to document structural changes indicative of glaucoma. In recent years, however, optical coherence tomography (OCT) has taken over as a more precise and objective tool in the diagnosis and monitoring of glaucoma. OCT relies on near-infrared light to obtain a cross-sectional image of the retina and optic nerve head. Different layers of the retina can be isolated in the scan, as there is an increased reflectivity in the nerve fiber layer and the plexiform layers and decreased reflectivity in the cell and nuclear layers. OCT performs a retinal nerve fiber layer (RNFL) analysis by measuring the RNFL thickness at a circle, 3.4–3.5 mm in diameter, centered around the optic nerve head. In the presence of a glaucoma, areas of RNFL loss are seen, typically in the superotemporal and inferotemporal regions of the circle. The OCT compares the RNFL thickness pattern with that of a normative database of the same age group and flags any areas below the reference range with a color-coded scheme. The OCT is a powerful tool in the early detection of glaucoma, as RNFL thinning can be seen, even before peripheral visual field loss occurs. Furthermore, when serial OCT scans are taken, the RNFL thickness of each region can be plotted on a trend line to assess for disease progression over time.

OCT is a desktop device only available in clinic. Companies are working to develop a portable version, but the closest to that at this time is the Envisu C2300 (Leica; Wetzlar, Germany), which consists of a handheld OCT scanhead attached to a cart of equipment. The Envisu C2300 is particularly useful for patients who cannot sit up to reach a desktop device. In the future, a portable home OCT machine for glaucoma patients may be available, given that Notal Vision (Manassas, VA, USA) has developed one for the detection of neovascular age-related macular degeneration (AMD) progression at home.

### 2.7. Visual Field

Glaucoma is known as the “sneak thief of sight” because of its tendency to affect the peripheral visual field first. As a result, many patients do not realize that they have glaucoma until the visual field loss encroaches centrally. In clinic, standard automated perimetry (SAP) is performed to detect visual field loss. The patient places his/her head on a chinrest in front of a white bowl. While fixating on a target, he/she is instructed to push a button whenever a spot of light is seen. The machine will display light stimuli at various locations and intensities. In the end, the machine will map out spots that the patient did significantly worse relative to his/her age group.

Because SAP is a large device only available in clinic, it cannot be used in a screening program outside or at home. Alternatives of SAP include online perimetry performed on a computer, tablet, or with virtual reality glasses.

#### 2.7.1. Peristat Online Perimetry

This is a visual field test accessed at keepyoursight.org and is performed on a computer with a 17-inch or larger monitor. In the beginning, the patient is asked to adjust his/her distance from the monitor until a flashing light temporal to the fixation point disappears (i.e., enters the blind spot). The patient is then asked to fixate at a central point. Stimuli are presented at various locations across a 24-degree horizontal by 20-degree vertical field with various intensities, and the patient is asked to push the spacebar whenever one is seen. The test takes less than 5 min per eye. Similarly to SAP, the Peristat test generates a report with reliability indices and a grayscale visual field image; the results are emailed to the doctor who ordered the test.

A prospective study [57] comparing Peristat Online Perimetry with Humphrey Visual Field 24-2 test reported Spearman rank correlations ranging from 0.55 to 0.77 for abnormal points in both tests. Peristat Online Perimetry demonstrated a high diagnostic ability, with the AUC ranging from 0.77 to 0.81 for mild glaucoma and 0.85 to 0.87 for moderate to severe glaucoma.

#### 2.7.2. Melbourne Rapid Fields (MRF)

This is a web-based program that relies on a touchscreen tablet. Voice prompts of multiple languages are available. The subject is asked to sit 33 cm away from the screen and fixate at a crosshair target. There is a square area that the subject is supposed to tap whenever a stimulus is seen. The stimuli are presented as dots of various intensities in different locations, similar to SAP. When using a small tablet, the fixation target may shift to a corner of the screen to widen the visual field area tested (to up to 30 degrees from fixation). The MRF test takes approximately 3–4 min per eye, which is significantly shorter than the Humphrey Visual Field 24-2 SITA-standard program (average 6–7 min per eye) [58,59]. After completion of the MRF test, a report is generated, showing the reliability indices, the sensitivity value of each spot, the total deviation map, the pattern deviation map, and the visual field gray scale. Multiple studies have shown that MRF has a low retest variability and high correlation with the Humphrey Field Analyzer [58,59,60,61].

#### 2.7.3. Virtual Reality Headsets

In recent years, virtual reality (VR) systems have become available to the public for entertainment, especially video gaming. VR headsets have gyroscopes that detect head movement and gaze trackers, allowing the user to immerse into a 3D virtual environment that shifts according to his/her head and eye movements. This technology is particularly useful in visual field testing because it eliminates the problem of fixation loss. The accuracy of a conventional visual field test relies on the subject to fixate at the target for the duration of the test. This is no longer necessary with a VR system, because the stimulus position can be adjusted based on the change in fixation. Tsapakis et al. [62] had 20 patients use virtual reality glasses hooked up to a computer. They ran a software that used a fast-threshold 3-decibel step staircase algorithm to test 52 points scattered across 24 degrees of visual field from fixation. The patients were asked to click the mouse whenever a stimulus was seen. This VR visual field test had a high correlation coefficient of 0.808 when compared with the Humphrey Visual Field. VR visual field systems that are commercially available include the Advanced Vision Analyzer (Elisar; New City, NY, USA), the C3 Field Analyzer (Remidio; Glen Allen, VA, USA), the PalmScan VF2000 Visual Field Analyzer (Micro Medical Devices; Calabasas, CA, USA), Virtual Field (Virtual Field; New York, NY, USA), VirtualEye Perimeter (BioFormatix; San Diego, CA, USA), VisuALL (OllEyes Inc, Summit, NJ, USA), and Vivid Vision Perimetry (Vivid Vision, San Francisco, CA, USA).

In addition to fixation loss, current visual field tests rely on patients to minimize the false-positive rate (meaning hitting the clicker when there is no stimulus presented) and the false-negative rate (meaning not hitting the clicker when the stimulus should be seen, based on previous responses). To eliminate the “human factor” of visual field testing, a VR headset called NGoggle was developed to detect multifocal steady-state visual-evoked potentials when a stimulus is presented. The headset consists of a wireless electroencephalogram, an electrooculogram, and a head-mounted display. In a study where glaucoma was diagnosed based on stereo photographs of the optic discs, Nakanishi et al. [63] found that the NGoggle had a higher AUC (0.92) than SAP mean deviation (0.81), SAP mean sensitivity (0.80), and SAP mean pattern standard deviation (0.77), suggesting that NGoggle may be better at detecting glaucoma than SAP. A VR headset that can detect visual-evoked potentials may prove to be more accurate and efficient than the current gold standard of SAP, setting a paradigm shift in visual field testing.

### 2.8. Artificial Intelligence

In ophthalmology, deep learning in artificial intelligence (AI) has become a hot topic as it demonstrated remarkable accuracy in the detection of disease. Deep learning is a machine learning technique that uses multiple layers of an artificial neural network to extract high-level features from raw data and generate an output. This design is inspired by how neurons connect with each other in the human brain. In order for the machine to generate a highly accurate neural network, it needs to be fed a massive set of data that encompass all variations. To make the diagnosis of glaucoma and recommend the appropriate management plan via telehealth, the ophthalmologist takes into account the IOP, visual field test report, fundus photography, OCT, and other available test results. Having a specialist review the data may not be necessary in the future, as artificial intelligence technology becomes more powerful with the ability to self-learn such as a human.

A number of deep learning AI systems have been developed to diagnose glaucoma based on optic disc photographs. The European Optic Disc Assessment Study [64] compared the performance of the Pegasus v1.0 (Visulytix Ltd., London, UK) AI software with that of ophthalmologists and optometrists in diagnosing glaucoma from stereoscopic optic disc photographs. Pegasus was able to diagnose with an accuracy of 83.4%, which was statistically similar to the accuracies of ophthalmologists (80.5%) and optometrists (80%). Eyes that truly had glaucoma were identified by glaucoma specialists who saw reproducible visual field scotomas that matched the appearance of the optic discs. Several other studies demonstrated that certain deep learning parameters can achieve high accuracy with AUC > 0.9 and sensitivity and specificity levels > 90%; false-positive and false-negative results were commonly due to pathologic myopia [65,66,67,68,69,70]. Even with the use of different fundus cameras, deep learning artificial intelligence was able to achieve an AUC > 0.9, provided that image augmentation was performed [71]. Al-Aswad et al. [72] used data from the Singapore Malay Eye Study to determine how the Pegasus deep learning system performed compared with ophthalmologists in diagnosing glaucoma solely based on fundus photographs. They found that Pegasus outperformed five out of six ophthalmologists and took only 10% of the time the ophthalmologists did in diagnosing glaucoma. Remarkably, Medeiros et al. [73] showed that by training a deep learning algorithm to match disc photographs with OCT RNFL scans, the machine was able to predict the average RNFL thickness based on the fundus photograph with a high Pearson correlation coefficient of 0.832 and a mean difference of 7.39 microns. In fact, when deep learning artificial intelligence was used on fundus disc photographs taken over time, it was able to identify eyes with worsening glaucoma based on a decreasing predicted RNFL thickness [74,75].

In addition to the analysis of fundus photographs, deep learning artificial intelligence can be trained to diagnose glaucoma based on OCT RNFL and the ganglion cell-inner plexiform layer (GCIPL) scans with high accuracy (AUC > 0.9) [76,77,78,79,80,81]. In fact, one study showed that a deep learning model trained with OCT images outperformed SAP and mean circumpapillary RNFL thickness in detecting glaucoma [76]. If the deep learning algorithms are exposed to OCT nerve images paired with visual field data, the machine is able to predict visual field parameters accurately [82,83].

Deep learning can also play a role in diagnosing angle closure based on OCT anterior segment images. Fu et al. [84] developed a deep learning system to detect angle closure and tested it on 8270 OCT anterior segment images (of which 895 had angle closure as classified by clinicians). The system achieved an AUC of 0.96 with a sensitivity of 0.90 and specificity of 0.92. Xu et al. [85] applied deep learning methods on 4036 OCT anterior segment images (of which 2093 had closed angles) in the Chinese–American Eye Study and found that the ResNet-18 classifier achieved an AUC of 0.952.

Machine learning of visual field data may have utility in diagnosing pre-perimetric glaucoma. Asaoka et al. [86] reported that a deep feed-forward neural network classifier had an AUC of 92.6% in diagnosing pre-perimetric glaucoma based on Humphrey Visual Field 30-2 data. In addition to diagnosis, deep learning has been shown to predict future visual field progression. Wen et al. [87] used various deep learning algorithms on more than 30,000 Humphrey Visual Field 24-2 reports and found that the Cascade-Net5 performed the best in forecasting visual fields up to 5 years later, with a pointwise mean absolute error (2.47 dB), significantly less than that of the rate of progression linear models (3.77–3.96 dB) and the pointwise regressed linear model (3.29 dB). Yousefi et al. [88] reported that machine learning analysis detected visual field progression earlier (at 3.5 years) than global (at 5.2 years), region-wise (at 4.5 years), and point-wise (at 3.9 years) linear regression analyses.

## 3. Setups for Telehealth Programs

As discussed previously, telehealth can serve three purposes: (1) screen for glaucoma, (2) evaluate the severity of glaucoma to determine the treatment plan, and (3) to monitor disease progression. How each of these purposes can be achieved should depend on the equipment/facilities available, the patient population (the prevalence of certain types of glaucoma can vary), and the socioeconomic and/or geographic barriers to face-to-face ophthalmologic care.

A number of telehealth screening programs have been implemented and can serve as templates tailored to the needs of the community. For example, the Philadelphia Telemedicine Glaucoma Detection and Follow-up Study [89] executed a program of which people at risk for glaucoma could be screened at a primary care practice or a Federally Qualified Health Center. At the first visit, the participant’s medical and family history were recorded. An ophthalmic technician used a nonmydriatic, portable fundus camera to take two fundus photographs (macula and optic nerve) and one anterior segment photograph per eye. The technician measured IOP using the iCare tonometer. If the IOP was ≥30 mm Hg, the participant was referred to an ophthalmologist immediately. All information obtained was sent to glaucoma and retina specialists for reading. If a participant had an IOP of 22–29 mm Hg, an abnormal finding (such as a suspicious optic nerve appearance), or an unreadable image, he/she were contacted to schedule an eye examination at the same primary care practice or health center within 6 months. At visit two, the subjects underwent a slit lamp examination by a glaucoma specialist, along with SAP. The equipment was brought in by a community outreach van. Based on the assessment at this visit, follow-up testing, appointment, or treatment was recommended. This screening program was conducted over 5 years. Of the 902 people screened, 37% had an abnormal image, 17.2% had an unreadable image, and 6.9% had ocular hypertension; therefore, 59.4% were asked to attend visit 2. Of the people asked to attend visit 2, 64.7% showed up. Of those who showed up, 10.9% had glaucoma, 7.2% had ocular hypertension, and 45.8% were glaucoma suspects. Taken together, 24.6% of the original 902 people screened had glaucoma, ocular hypertension, or were glaucoma suspects in this urban, multiethnic population.

Similar to the Philadelphia screening program, the Manhattan Vision Screening and Follow-up Study in Vulnerable Populations (NYC-SIGHT) [90] is a community-based screening program to be conducted for 5 years, specifically for residents in New York City Housing Authority developments. Because of the COVID-19 pandemic, participants will have medical history obtained and a visual function questionnaire asked over the phone and will be screened for COVID-19 symptoms before being allowed at the community screening site. At the screening, visual acuity check, IOP measurement, and nonmydriatic fundus photography are performed. Participants who fail the vision screening will be scheduled to see an optometrist on-site for a refraction and a nondilated eye examination by a portable slit lamp and a direct ophthalmoscope. Participants with a high IOP, an abnormal fundus image, or a concerning examination finding will be referred to an ophthalmologist in the clinic.

The Michigan Screening and Intervention for Glaucoma and Eye Health Through Telemedicine (MI-SIGHT) [91] and the Alabama Screening and Intervention for Glaucoma and Eye Health Through Telemedicine (AL-SIGHT) [92] programs are designed to be more comprehensive at the first visit. Similarly to the Philadelphia program, federally qualified health centers are used, and people with specific risk factors for glaucoma are eligible. In the Alabama program, an ophthalmic technician checks the visual acuity, performs auto-refraction, measures IOP with iCare rebound tonometer, and takes images using a combined OCT and fundus camera machine (Maestro2, Topcon Medical Systems, Oakland, NJ, USA) and a smartphone with an adapter (D-Eye retinal camera), and performs visual field testing using the Humphrey Visual Field Analyzer and Melbourne Rapid Fields application on a tablet. In the Michigan program, an ophthalmic technician checks the visual acuity and performs refraction, assesses the iridocorneal angle with penlight, assess ocular motility, measures IOP with iCare tonometer, and dilates the participant if IOP < 30 mm Hg and/or angles are not narrow. Fundus photographs and OCT RNFL images are obtained when the pupils are dilated. In both programs, the data are sent to an ophthalmologist for review in order to determine the appropriate follow-up.

Rather than screening the community for glaucoma, a telemedicine program [93] in Northern Alberta served as a glaucoma consult service. Patients seen by an optometrist, ophthalmologist, or family physician were referred to the program if they had risk factors for glaucoma or suspicious-looking optic discs or visual field test. At each office, a tonometer, corneal pachymeter, visual field machine, and a retinal camera were available for use by technicians. A glaucoma specialist at the University of Alberta then reviewed the data remotely and gave recommendations for management and follow-up.

In addition to screening for glaucoma, telemedicine can be used to monitor for the development of glaucoma. The Kaiser Permanente Eye Monitoring Center conducted a 2-year telemedicine program [94] to monitor low-risk glaucoma suspects. Each year, a technician checked the visual acuity, measured the IOP using a handheld applanation tonometer, and took OCT RNFL images at a local ophthalmology clinic. Different from other telemedicine programs, the data were sent to a trained technician first, rather than a glaucoma specialist. If there was a decline in visual acuity of at least two lines, an IOP elevation ≥ 5 mm Hg, or a significant change in the RNFL thickness in the superotemporal or inferotemporal region (defined as ≥10-micron reduction or transition into the abnormal red range), the technician would send the patient data to a glaucoma specialist for review remotely. Of the 225 glaucoma suspects enrolled in the program, five were referred for examination by an ophthalmologist due to concern for progression on OCT. Of those five patients, two were started on glaucoma medications. This program demonstrated that telemedicine is a viable option for monitoring glaucoma suspects and can capture the small number of patients who develop glaucoma and need treatment.

Telehealth programs can be used to monitor patients with an established diagnosis of glaucoma as well. Rutgers New Jersey Medical School conducted a program [95] on patients who had glaucoma or were glaucoma suspects. The patients went through the tele-glaucoma setup in the following order: (1) medical history intake; (2) IOP measurement with puff tonometer; (3) auto-refraction; (4) OCT imaging of the iridocorneal angle (and central corneal thickness measurement), cup/disc ratio, RNFL, and ganglion cell complex; (5) nonmydriatic color photography of the anterior segment and fundus, as well as auto-fluorescence imaging of the fundus. A glaucoma specialist then reviewed the data remotely and gave recommendations on management and follow-up. To compare the accuracy of the data and assess the program with a clinical examination, the subjects underwent a comprehensive eye examination on the same day by an ophthalmologist. IOP was measured by Goldmann applanation and the slit lamp was used to examine the anterior segment and fundus. OCT and visual field testing were performed under the discretion of the ophthalmologist. When comparing the tele-glaucoma program with the clinical examination, there were strong correlations in IOP measurements and cup/disc ratios. The recommended follow-up time was shorter for the tele-glaucoma program (2.7 months vs. 3.9 months). The clinical examination was better at identifying exotropia, iridotomy, iris neovascularization, and trabeculectomy. The tele-glaucoma program was better at identifying narrow angles, age-related macular degeneration, macular edema, diabetic retinopathy, retinal vein occlusion, choroidal nevus, and splinter hemorrhages.

A similar study [96] in London compared a “virtual clinic” staffed by an ophthalmic nurse versus an examination by an ophthalmologist for patients with open angle glaucoma. In the nurse clinic, a technician checked visual acuity, conducted SAP, took fundus photos, and performed scanning laser ophthalmoscopy with Heidelberg Retina Tomography. The nurse performed Goldmann applanation tonometry and a slit lamp examination of the anterior segment. The patient then was examined by an ophthalmologist and the assessment was recorded. One year later, the same ophthalmologists who took part in the study were asked to review the data from the nurse “virtual clinic” a year ago and classify whether the patient was stable or unstable based on just these data. The study found that 3.4% of patients were misclassified as “stable” by review of the “virtual clinic” data when in fact they were “unstable” according to the in-person assessment by the ophthalmologist. The authors concluded that 3.4% was an acceptably low misclassification rate and that a “virtual clinic” run by ophthalmic nurses can be a viable option for managing relatively stable glaucoma patients.

A review of these glaucoma telehealth programs shows that a variety of setups can be used to screen for glaucoma and monitor for disease progression. At the bare minimum, a technician should record the patient’s medical history, visual acuity, IOP, and take a fundus photograph. Technological advances have allowed IOP measurements without the use of topical anesthetic and fundus imaging without pharmacologic pupillary dilation. SAP to detect visual field scotomas and OCT to detect structural nerve fiber layer loss can provide additional valuable data, but these bulky machines are unlikely available outside of the ophthalmology clinic setting. As deep learning artificial intelligence technology matures, fundus imaging may be all that is needed to accurately predict RNFL thickness and visual field loss. Artificial intelligence will play a significant role in reducing the amount of equipment required for glaucoma screening and monitoring through telehealth. A summary of the components of a glaucoma telehealth examination is listed in Table 1. Even at its current state, without reliance on artificial intelligence, telehealth has shown to be cost-effective. An analysis of remote glaucoma screening in rural Alberta, Canada revealed that teleglaucoma costs an average of CAD 867 per patient, which was dramatically less than the average CAD 4420 per patient for in-person screening [97]. In order to control healthcare costs while providing access to care, especially in rural regions, telehealth will become an important tool in the screening and monitoring of chronic diseases such as glaucoma.

## 4. How the Coronavirus Pandemic Shaped Telehealth

In December 2019, a novel respiratory illness COVID-19 emerged in Wuhan, China. Because of the disease’s highly contagious nature, it quickly spread globally, and a pandemic was declared by the World Health Organization on 11 March 2020. Governments worldwide imposed lockdowns to stop the spread of disease, as COVID-19 overwhelmed hospital systems with vast numbers of people requiring ventilators. In the United States, state governments issued stay-at-home orders and social distancing guidelines. People were asked to work from home and to avoid venturing outside except for essential activities. On 18 March 2020, the American Academy of Ophthalmology (AAO) recommended the cessation of elective surgery and routine clinic visits to protect patients from catching COVID-19 and to conserve personal protective equipment (PPE).

Because many ophthalmology practices closed their offices, telehealth through video visits became a necessary way for patients to see their doctors. In the United States, the Centers for Medicare and Medicaid Services (CMS) relaxed the requirements to bill for telehealth visits; thus, allowing practices to be reimbursed for remote patient care. Many practices implemented a telemedicine program for the first time and had to develop protocols to address patient needs virtually. Saleem et al. [98] depicted a workflow diagram as a reference to implement an ophthalmology telemedicine program. Essentially, the front desk staff reaches out to patients who had their appointments canceled and offers them a telephone or video visit for non-urgent problems. If the patient describes an issue that appears to be an emergency, the physician is contacted to determine whether the problem can be addressed remotely or the patient must be examined in person.

A major hurdle in managing glaucoma patients through video visits is that glaucoma, for the most part, is an asymptomatic disease, unless there is a substantial increase in IOP causing eye pain or rapid visual field loss causing noticeable constriction in vision. A video visit does not allow for IOP measurement, visual field testing, or the visualization of the optic nerve. A crude method for the patient to estimate IOP is via finger palpation on the eye through the eyelid. A more accurate way than digital palpation is for the patient to use the iCare HOME rebound tonometer on him/herself. The tonometer is easy to use, comfortable, and requires no topical anesthetic. Because the device is expensive, companies such as MyEYES (myeyes.net) and Enlivened (enlivened.com) offer rentals for a fee. Patients are taught how to use the device and borrow it for one or more weeks. The downside, however, is that the IOP readings are not displayed; the patient must return the device to the office to extract the IOP diurnal curve. An alternative to using the iCare HOME is to wear the Sensimed Triggerfish^®^ Contact Lens, which makes automated corneoscleral dimensional measurements for 24 h. However, the patient is required to have a circular antenna taped around the eye, wear a recording device hanging from the neck, and return to the office the next day to extract the diurnal curve. A new contact lens being developed in South Korea allows for convenient IOP monitoring using a smartphone. Implantable devices such as the Eyemate^®^ and Injectsense can provide IOP monitoring as well. If there is concern for visual field progression, the patient can use the computer-based Peristat Online Perimetry, the tablet-based Melbourne Rapid Fields program, or virtual reality perimetry to generate a visual field report and send it to the physician.

As pandemic lockdown restrictions loosened, ophthalmology practices reopened with the implementation of new protocols for the safety of the patients and staff members. Vinod et al. [99] described methods that practices used to enforce social distancing and enhance safety, such as limiting the number of appointments, rearranging chairs in the waiting rooms, asking patients to remain in their cars outside the clinic until they are called, mandating everyone to wear masks, and installing large breath shields on slit lamps. However, some patients are still uncomfortable with in-person examinations and prefer telehealth until the pandemic ends.

## 5. Conclusions

**Figure 1:**
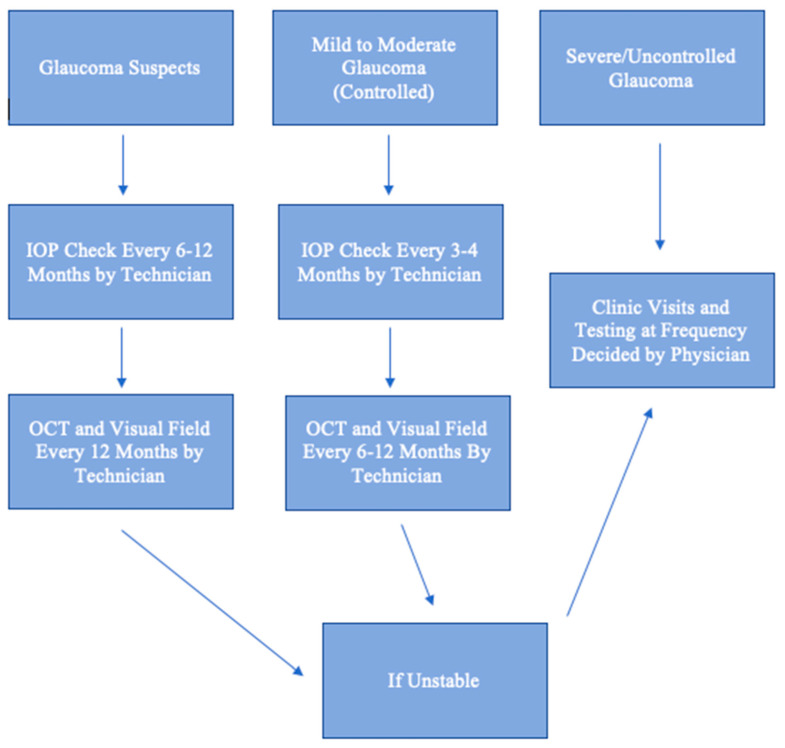
Algorithm for Remote Monitoring. IOP: intraocular pressure. OCT: Optical coherence tomography.

As demand for glaucoma care increases, there will be a need for telehealth. Just as radiologists review scans remotely, ophthalmologists can review results and risk-stratify patients. A glaucoma suspect can be monitored remotely, provided that one has access to an OCT or visual field machine yearly. A patient with well-controlled mild to moderate glaucoma can also be monitored remotely if one has IOP measurements performed regularly and that an in-person dilated examination is performed annually. A patient with uncontrolled or severe glaucoma should have face-to-face visits, as there is much less room for error and a high likelihood of needing laser or surgical procedures. This algorithm for remote monitoring is illustrated in Figure 1. Essentially, face-to-face examinations can be limited to confirmation of diagnosis, management of patients with uncontrolled or severe glaucoma, and patients with new, concerning ocular symptoms. The telehealth approach is cost-effective and can increase patient satisfaction by decreasing waiting time during visits. Telehealth is particularly beneficial for patients in rural areas who have limited access to care and in the setting of a pandemic, when social distancing is enforced and the number of appointments is severely limited to reduce disease spread. Deep learning artificial intelligence will play an increasing role in the diagnosis and management of glaucoma using data extracted from telehealth.

## Figures and Tables

**Table 1:** Components of a glaucoma telehealth examination.

	Utility	Disadvantages
Visual Acuity	Changes can be due to a new central scotoma, refractive error, cataract, and other ocular pathologies.	Glaucoma typically presents with peripheral visual field loss which visual acuity does not assess. Only very advanced glaucoma affects visual acuity.
Intraocular Pressure (IOP)	A very important parameter to assess the efficacy of treatment and a major risk factor for disease progression.	Goldmann applanation, the gold standard for measuring IOP, is only performed in clinic. Portable tonometers can significantly differ from Goldmann measurements for IOPs outside the normal range.
Anterior Segment Photography	In lieu of the slit lamp examination, the camera can capture abnormalities of the external and anterior parts of the eye.	The camera may miss subtle pathologies such as a pigment deposition on the corneal endothelium or iris neovascularization. In addition, it cannot capture the anterior chamber cell and flare.
Iridocorneal Angle Imaging	Identifies eyes with anatomic narrow angles at risk for acute angle closure glaucoma.	Angle camera devices and UBM require instillation of topical anesthetic.
Fundus Photography	Captures images of the optic nerve head and macula. Progressive cupping of the optic nerve is a sign of uncontrolled glaucoma.	Although many cameras do not require pharmacologic dilation, the brightness and resolution of the images may be affected by pupil size.
Ocular Coherence Tomography (OCT)	Measures retinal nerve fiber layer thickness using near-infrared light. A reference database is available for comparison. An abnormally thin nerve fiber layer or progressive thinning is a sign of uncontrolled glaucoma.	The device is not portable and is only available in clinic.
Visual Field	Testing is important to detect early peripheral visual field loss and to monitor for expansion of the scotoma. The amount of visual field loss determines the severity of disease and plays a role in setting the target IOP.	Traditional standard automated perimetry is not portable and is only available in clinic. Visual field monitoring at home can only be performed using a web-based program on a computer, a tablet, or with virtual reality glasses.
Artificial Intelligence (Deep Learning)	By self-learning via an artificial neural network, the technology has demonstrated remarkable accuracy in diagnosing glaucoma and monitoring for disease progression using fundus images, OCT, and visual field.	It is still under development and not available to the public yet.

## Data Availability

Not applicable.

## Consultant

Eyenovia: ReNetX Bio, SmartLens. Equity Interest: Ocutrx, SmartLens.
